# Rift Valley Fever Virus Transmission During an Unreported Outbreak Among People and Livestock in South-Central Tanzania

**DOI:** 10.3390/v17101329

**Published:** 2025-09-30

**Authors:** Robert D. Sumaye, Ana Pérola D. Brandão, Frank Chilanga, Goodluk Paul, Grace W. Mwangoka, Woutrina A. Smith, Abel B. Ekiri, Christopher Kilonzo, Solomon Mwakasungula, George Makingi, Amina A. Kinyogori, Walter S. Magesa, Aziza J. Samson, Catherine Mkindi, Peter Pazia, Feisal Hassan, Thabit A. Mbaga, Robinson H. Mdegela, Honorati Masanja, Deborah Cannon, Aridith Gibbons, John D. Klena, Joel M. Montgomery, Stuart T. Nichol, Lucija Jurisic, Alexandre Tremeau-Bravard, Hezron Nonga, Jamie Sebastian, Saba Zewdie, Leah Streb, Anna C. Fagre, Nicholas A. Bergren, Daniel A. Hartman, David J. Wolking, Rebekah C. Kading, Jonna A. K. Mazet, Brian H. Bird

**Affiliations:** 1 Ifakara Health Institute, Dar es Salaam P.O. Box 78 373, Tanzania; rsumaye@ihi.or.tz (R.D.S.);; 2 One Health Institute, School of Veterinary Medicine, University of California Davis, Davis, CA 95616, USA; abrandao@ucdavis.edu (A.P.D.B.);; 3 Department of Veterinay Medicine and Public Health, Sokoine University of Agriculture, Morogoro P.O. Box 30 007, Tanzania; 4 Centers for Disease Control and Prevention, Viral Special Pathogens Branch, Atlanta, GA 30329, USA; 5 Center for Vector-Borne Infectious Diseases, Colorado State University, Fort Collins, CO 80523, USA

**Keywords:** Rift Valley fever, epidemiology, disease surveillance, virus transmission, zoonoses, inter-epidemic transmission, endemic, arboviruses, One Health, Tanzania, hemorrhagic fever

## Abstract

Rift Valley fever (RVF) is a re-emerging vector-borne zoonotic disease that causes outbreaks in humans and animals across Africa. To better understand RVF at human–animal interfaces, a prospective longitudinal survey of people, livestock, and mosquitoes was conducted from 2016 to 2018, in two regions of Tanzania, with distinct climatic zones (Iringa and Morogoro). Molecular and serological tools for testing (RT-qPCR and IgM/IgG ELISA) for RVF virus (RVFV) were used to assess infection and exposure in people and animals. Mosquitoes were collected quarterly from 10 sentinel locations. In total, 1385 acutely febrile humans, 4449 livestock, and 3463 mosquito pools were tested. In humans, IgM seroprevalence was 3.75% (n = 52/1385), and overall seroprevalence (IgM and/or IgG positive) was 8.30% (n = 115/1385). People from Iringa had a higher exposure risk than those from Morogoro (aOR 2.63), and livestock owners had an increased risk compared to non-owners (aOR 2.51). In livestock, IgM seroprevalence was 1.09%, while overall seroprevalence was 10.11%. A total of 68.4% of herds had at least one seropositive animal. Sentinel animal follow-up revealed that the probability of seroconversion was significantly higher in Morogoro. Low-level RVFV RNA was detected in 8 human and 22 mosquito pools. These findings indicate active transmission among vectors, livestock, and people during the study period, highlighting the need for One Health surveillance approaches for RVFV and other arboviruses.

## 1. Introduction

Rift Valley fever (RVF) is a high-consequence zoonotic disease that can cause periodic large-scale outbreaks in humans and livestock across Africa and parts of the Arabian Peninsula. First reported in the 1930s in Kenya [1], the disease is caused by Rift Valley fever virus (RVFV, Family: *Phenuiviridae*, Genus: *Phlebovirus,* Species: *Phlebovirus riftense*) [2,3,4]. Livestock animals (sheep, cattle, and goats) are highly susceptible to infection and develop severe and often fatal disease accompanied by widespread fetal loss that is economically devastating to livestock-keeper livelihoods [5]. Humans typically develop mild to moderate self-limiting infections characterized by acute-onset fever, malaise, and myalgia/arthralgia. In a minority of RVFV-infected individuals, the disease can progress to severe hepatitis, retinitis, and in some cases, a delayed-onset neurological syndrome [6,7]. Recent reports suggest that RVFV may contribute to increased fetal demise and spontaneous abortion among infected women [8,9]. Among hospitalized patients, case fatality ratios can exceed 30 to 50% [10,11].

During inter-epidemic periods in some regions, transovarial transmission among *Aedes* spp. Flood-water mosquitoes appear to contribute to the long-term persistence of the virus in endemic areas [12]. During outbreak cycles, a wide variety of mosquito species can become infected after feeding on viremic animals [13,14]. Transmission to humans can be from an infected mosquito bite, or more likely through contact with infected animals and RVFV-contaminated body fluids, aborted materials, tissues, milk, and other animal products [15,16,17].

In Eastern Africa, regional outbreaks of RVF have historically occurred in 5–15 year cycles, typically following unusually heavy rainfall and sustained flooding [18,19]. In these regions, the RVFV epidemic pattern has been closely linked to El Niño/Southern Ocean oscillation patterns, leading to climatic conditions accompanied by long duration and intense periods of high precipitation [18,20]. While these precipitation events are associated and can be helpful predictive early-warning tools for the occurrence of large-scale outbreaks, occasionally outbreaks do not occur or are not recognized despite optimal environmental conditions. The exact underlying factors precipitating the initiation of regional-scale RVFV outbreaks remain to be fully elucidated.

RVF outbreaks have been reported across diverse ecological zones from highly arid areas in Western Africa [21,22,23,24], tropical Central Africa, the East African Rift Valley [23,24,25,26,27,28,29,30,31,32], Madagascar/other Indian Ocean islands [33,34], and, beginning in the year 2000, in the Arabian Peninsula [35,36]. RVFV and its mosquito vectors have repeatedly crossed international and geographic borders, and recent serological evidence suggests that RVF is continuing to expand its geographical range further into North Africa and potentially to the Mediterranean region [37,38,39].

While large epizootics are a hallmark of RVFV activity in Eastern and Southern Africa, the epidemiology and disease transmission dynamics seem to be changing. Regular seroconversions of animals and people have been documented in the absence of large-scale classical outbreaks in Uganda [32], Kenya [40,41,42], Tanzania [43,44,45], Zambia [46,47], and South Africa [48,49,50]. The presence or absence of ongoing low-level endemic transmission in discrete focal habitats that help initiate regional outbreaks may explain, in part, some of the discrepancies between climatic model predictions and the occurrence of the regional devastating outbreak events. The emergence of atypical patterns of transmission and novel RVFV genotypes may also contribute to the apparently changing pattern of RVF epidemiology across much of Africa [32,51]. Regardless of the exact ecological mechanisms, it is clear that the transmission dynamics of RVF during epidemics and inter-epidemic periods are complex.

To investigate the potential for inter-epidemic period transmission of RVFV among livestock, vectors, and humans, an integrated One Health approach was taken to conduct multi-year concurrent sampling of humans, livestock, and vectors in two distinct climatic zones in south-central Tanzania. Longitudinal surveillance was conducted for 28 months in both the Ruaha ecosystem (high elevation and drier conditions—primarily located in the Iringa region—with a high level of human–livestock–wildlife interactions), and the Kilombero ecosystem (low elevation and wetter conditions—primarily in the Morogoro region—that typically experiences large-scale annual flooding).

## 2. Materials and Methods

### 2.1. Study Design and Areas

This One Health longitudinal study was conducted to investigate RVFV exposure in humans, livestock, and mosquito vectors, along with associated risk factors, from March 2016 to May 2018. The study was conducted in two regions in south-central Tanzania, including the Ruaha National Park and River ecosystem (Iringa region) and the Kilombero River Valley ecosystem (Morogoro region). The Ruaha ecosystem spans one district (Iringa Rural) that comprises the highlands of the Rift Valley, between 900 and 1200 m above sea level, and it borders wildlife-protected areas: the Ruaha National Park to the west and a mountain escarpment to the east. The climate is semi-arid, with bimodal rainfall usually between December and March, receiving an average annual rainfall of 500–600 mm and temperatures ranging between 20 and 25 °C [52]. Iringa Rural District had an estimated 155,355 cattle, 103,429 goats, and 36,760 sheep during the time of completion of this study [52]. The Kilombero River Valley is a seasonally inundated flood plain that spans two districts of Ulanga and Kilombero. The valley is about 300 m above sea level, located between the forested escarpment of the Udzungwa mountains on the north-western side and the Mahenge mountains on the south-eastern side. The valley receives an average annual rainfall of 1200–1800 mm, usually from December to January and March to May. Temperatures range between 25 °C and 32 °C. The inhabitants of the Kilombero valley engage mainly in smallholder farming, fishing, and livestock keeping [53]. During the completion of these studies, Kilombero and Ulanga districts had an estimated 200,156 cattle, 51,508 goats, and 40,719 sheep kept by agro-pastoralists and pastoralists under traditional farming systems [54].

### 2.2. Permissions and Regulatory Approvals

This study was approved by the Institutional Review Board (IRB) and Institutional Animal Care and Use Committee of University of California, Davis (protocols 785118 and 19271, approved 7 March 2015 and 5 August 2015, respectively), the Ifakara Health Institute (IHI) IRB (No: 16-2015, approved 17 June 2015), and the National Health Research Ethics Committee (NatHREC) of the Tanzania’s National Institute for Medical Research (NIMR; permit number NIMR/HQ/R.8a/Vol.IX/2062, approved 12 November 2015). Research approvals were also obtained from the Tanzania Commission for Science and Technology (COSTECH; permit number: 2016-323-NA-2006-179 and RCA 2006/79, approved 18 August 2015). In each community, an informational meeting was held for community leaders, stakeholders, and other community members to seek local permissions prior to initiating any work.

### 2.3. Human Participant Enrollment

**Figure 1:**
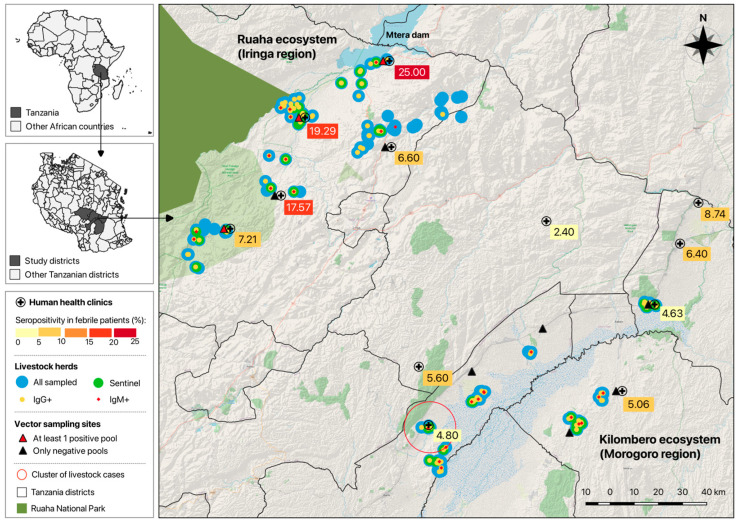
Study area indicating the locations for human, animal, and vector sampling, as well as results for human seropositivity by health clinic, herd serostatus, and vector sites with positive pools and cluster of livestock cases.

Twelve health clinics, 7 from Iringa region and 5 from Morogoro region (Figure 1), were selected from those that served the communities with reported wildlife–livestock–human interactions and where previous RVFV outbreaks and possible inter-epidemic transmission were known to occur [43,53]. Inclusion criteria were patients with fever (axillary body temperature ≥ 37.5 °C) from selected health clinics, who were residents in the catchment area villages, and who were ≥5 years old. For each participant, written informed consent was obtained. For minor participants (5 to 17 years old), parents or legal guardians provided written consent, and children aged 7 to 17 also provided assent to participate. Illiterate participants had their consent witnessed by another literate person. Each study enrollee was assigned a unique participant ID upon enrollment, used for subsequent data tracking and analyses. In addition, a structured questionnaire was administered at the time of enrollment to assess potential risk factors for exposure to RVFV (Supplementary Materials File S1).

### 2.4. Livestock Enrollment Framework

The livestock research was subdivided into 3 major components: (1) cross-sectional bio-surveillance of 193 livestock herds, (2) establishment of a sentinel network of RVFV seronegative cattle from the cross-sectional cohort, and (3) opportunistic reporting by livestock keepers in the study area to allow for sampling of aborted livestock fetal materials. Prior to sampling, the community and each animal herd owner were informed of the overarching study goals and of the potential risks associated with sampling activities. Consent was obtained from the head of the household or a representative involved in management decision-making regarding livestock before any animal-related sampling activities were performed.

#### 2.4.1. Cross-Sectional Livestock Study

Livestock herds (n = 193) were selected through a stratified random sampling approach from a sampling frame of pastoralists identified from previous research work in the same study area [53,55,56]. Selected herds included those with cattle and small ruminants (sheep and goats). The number of cattle sampled in each herd was dependent on herd size, with an additional 10 sheep and 10 goats sampled for a representative sample of these taxa groups from each selected herd. Cattle were individually marked with a numeric ear tag for identification and subsequent sentinel study inclusion. Individual animal demographic information, including sex, age, parity, RVF vaccination history, and relevant clinical features such as previous abortion for female animals, was recorded. Herd-level information about species composition and other risk factors for RVF transmission, such as current and past abortion cases, and recent introduction of animals in the herd, were also recorded.

#### 2.4.2. Sentinel Livestock Herd Study

After initial screening of all animals to determine both individual and herd seropositive status, herds were stratified into high, low, and negative herd-level status. From these, a proportion of seronegative animals were re-enrolled for quarterly sampling during the study period, to assess active seroconversion and RVFV transmission/exposure events.

### 2.5. Human and Livestock Sampling

Whole blood and serum samples for humans and livestock were collected into sterile cryovials for storage. Aliquots were added to either Trizol reagent (ratio 5:1 for specimen inactivation; ThermoFisher, Waltham, MA, USA) directly or stored without buffer in a liquid nitrogen dewar before shipping to the laboratory for further processing and testing. Malaria testing was performed using a malaria rapid diagnosis test kit (mRDT, SD-Bioline^TM^, Standard Diagnostics, Abbott Inc., Lake Forest, IL, USA).

### 2.6. Mosquito Biosurveillance

Vector mosquito sampling was conducted in 10 locations across the study region: five in the Ruaha ecosystem and five in the Kilombero ecosystem (Figure 1). In each location, five sentinel trapping sites were selected among households that participated in the cross-sectional livestock survey. Sampling was conducted quarterly from August 2016 to April 2018. During each round of sampling, the team set four traps in livestock-keeping households that were hung in a cattle/goat/sheep shed or in a bedroom with an occupied bed and a bednet. In addition, one trap was set in an outside environment in a nearby grazing area or forestry environment, baited with CO_2_ prepared from sugar and yeast fermentation [57]. All traps were operated for five nights. Mosquitoes were collected using miniature CDC light traps (John W. Hock Company, Gainesville, FL, USA). The following morning after each night of trapping, the traps were collected and the mosquitoes from each trap were prepared for sorting by anesthetizing them using the triethylamine-based cocktail FlyNap^®^ (Carolina Biological Supply, Burlington, NC, USA). Initial sorting of mosquitoes to specific genera was performed in the field; mosquitoes were then stored in liquid nitrogen dewars/shipper prior to shipping to the laboratory for morphological speciation [58,59]. Mosquitoes were pooled into microcentrifuge tubes (≤25 mosquitoes per pool) by collection date, trap location, species, sex, and blood meal status. A sample of fully blood-engorged mosquitoes from each trap was stored individually in standard 2.0mL microcentrifuge tubes (ThermoFisher, MA, USA) for blood meal analysis.

### 2.7. Laboratory Analyses

#### 2.7.1. Detection of RVFV Specific Antibodies

For humans, exposure status was determined through detection of anti-RVFV IgM and IgG antibodies using the enzyme-linked immunosorbent assay (ELISA) method as previously described [60,61,62]. All sera were rendered non-infectious by Cobalt 60 (Co_60_) gamma-irradiation (dose 5 million RADS) prior to testing. Briefly, the serum was diluted four-fold (100 to 6400), and optical density (OD) values were measured and reported as an adjusted SUM_OD_, indicating the sum of ODs across dilutions minus background ODs at each dilution. Specimens were considered positive if adjusted SUM_OD_ values exceeded the mean plus three standard deviations of known previously characterized negative control human specimens (non-vaccinated and non-exposed to RVFV individuals) run concurrently.

For livestock, all sera were heat inactivated (56 °C, 30 min in an immersion water-bath) and then tested for RVFV IgG antibodies using the ID Screen-Rift Valley Fever Competition Multispecies ELISA kit (ID vet, Grabels, France) and for IgM antibodies using the ID Screen-Rift Valley Fever IgM Antibody Capture ELISA kit (ID vet, Grabels, France), as per manufacturer instructions.

#### 2.7.2. RNA Extraction and Reverse Transcriptase Quantitative PCR (RT-qPCR)

Total RNA was extracted from human and animal whole blood or sera in Trizol (whole blood) using the Direct-zol RNA MiniPrep kit (ZYMO Research, Irvine, CA, USA) and the Viral Pathogen detection kit, Ambion-ThermoFisher, MA, USA) per the manufacturer’s instructions and as described [63]. Detection of the RVF virus genome was performed using one-step RT-qPCR as previously described [62]. RVF primers and a probe for detection of the L segment of the RVF virus were used: RVFL-2912 forward (5′-TGAAAATTCCTGAGACACATGG-3′), RVFL-2981 reverse (5′-ACTTCCTTGCATCATCTGATG-3′), and RVFL-probe-2950 (5′-CAATGTAAGGGGCCTGTGTGGACTTGTG-3′). Endogenous 18s rRNA was used as a negative control with 18s rRNA Universal Control primers–probe mix (Applied Biosystems-ThermoFisher, MA, USA), and commercially available RVF livestock vaccine (Smithburn, Onderstepoort Biological Products, Onderstepoort, South Africa) was used as a positive control. For each 96-well plate, a 25 μL reaction mix per sample was prepared for the RVF targets and contained 12.5 μL 2× master mix, 0.5 μL RT/Platinum Taq Mix, 0.5 μL forward primer (10 μM), 0.5 μL reverse primer (10 μM), 0.5 µL probe (5 μM), 0.05 μL Rox dye, 5.45 μL nuclease-free water, and 5 μL RNA template. On the same plate, another 25 μL reaction mix per sample was prepared for 18s targets and contained the same mix as described above, except the 18s primer-probe mix. The cDNA synthesis and qPCR were carried out in a CFX96 real-time PCR machine (BIO-RAD, Hercules, CA, USA) under the following conditions: Reverse Transcription (RT) performed at 50 °C for 30 min, RT inactivation/Taq activation at 95 °C for 2 min followed by PCR amplification (40 cycles) at 95 °C for 15 s and 60 °C for 45 s.

#### 2.7.3. RVF Virus Genome Detection in Mosquito Samples and Blood Meal Identification

Mosquito pools were homogenized into a 2 mL microcentrifuge (ThermoFisher, MA, USA) tube containing two 5 mm stainless-steel beads (Qiagen Inc., Valencia, CA, USA) and 1000 μL of VTM using the TissueLyser LT bead mill (Qiagen Inc., Valencia, CA, USA). The homogenate was clarified by centrifugation at 12,000 rpm for one minute, and the supernatant was inactivated in virucidal lysis solution to enable subsequent RNA extraction using the Direct-zol^TM^ RNA MiniPrep kit (Zymo Research, Irvine, CA, USA) according to the manufacturer’s instructions. RVF virus RT-qPCR was performed as described above, in a Step One PCR machine (Applied Biosystems, ThermoFisher, MA, USA). The vertebrate blood source of individual engorged mosquitoes was determined using a multiplex PCR assay targeting amplification of human, cow, dog, goat, and pig DNA [64].

### 2.8. Data Analysis and Mapping

All human demographic information was de-identified prior to analyses, after de-identificaiton data fields including sex, age, village of residence, relevant clinical signs/features, date of onset, date of presentation, date of enrollment, and date of sampling were utilized, which were recorded on the study participants’ screening form (Supplementary Materials File S1). The association between RVF seroprevalence and potential risk factors in people and livestock was evaluated by using a logistic regression model in R, version 4.1.2 (http://cran.r-project.org/, last accessed 1 September 2025). Variables were first assessed singly, and those significant at *p*-value < 0.2 were selected for inclusion into a multivariable model. A mixed effects logistic regression model was fit to both human and livestock data, with the health clinic and herd, respectively, set as a random effect to account for data dependence. Different models were compared using the Akaike information criteria (AIC). Therefore, variables selected for inclusion did not necessarily remain in the final multivariable model, as stepwise selection was applied based on model fit and statistical significance.

A cluster analysis was performed in SaTScan version 10.3 [65] to identify spatial clusters of RVF seropositivity in livestock. A purely spatial scan statistic was applied using the Bernoulli probability model and a circular scanning window, setting the maximum cluster size to 50% of the study population. Statistical significance was assessed using 999 Monte Carlo replications, and clusters with a *p*-value < 0.05 were considered significant. Spatial data was processed and visualized using QGIS software version 3.34.10-Prizren.

## 3. Results

A total of 1388 febrile human participants were enrolled at the 12 rural health clinics selected. From those, 1385 had complete data and were further analyzed. The livestock sampled were composed of 4449 animals, including 2300 cattle, 1193 goats, and 956 sheep, across the 193 herds selected. In general, livestock animals were reported to be clinically healthy at the time of sampling. Mosquitoes were collected and organized into 3463 pools. Laboratory results for each population by region are shown in Table 1. Location of health clinics, livestock herds, and vector sampling sites are shown in Figure 1, along with results on human seropositivity, herds’ serostatus, and vector sites with positive pools.

### 3.1. RVFV Detection, Patient Clinical Characteristics, and Risk Factors in Humans

From the 1385 participants with complete data, 89.1% were ≥11 years old, 69.3% were females, and 53.9% were from the Ruaha ecosystem (Iringa region). Apart from fever, other clinical symptoms included chills (87.9% of 1370), headaches (86.1% of 1379), malaise (83.4% of 1382), joint pain (72.2% of 1384), abdominal pain (70.0% of 1381), lack of appetite (57.7% of 1379), and muscle pain (57.7% of 1379). Twenty-five percent (25.0%) of 1384 study participants tested positive on a malaria rapid diagnostic test.

Recent exposure to RVF was 3.75% (95% CI: 2.84–4.92%), as determined by detection of RVFV-specific IgM antibodies in 52 febrile individuals. IgM-positive patients were detected in every month of the study period except for March and December, with 32 detected in the dry season and 20 in the wet season. Considering the detection of antibodies in both tests (IgG and/or IgM positive), a total of 115 individuals were seropositive. Thus, the overall RVFV seroprevalence in people was 8.30% (95% CI 6.90–9.88%). Of these, 78 (67.8%) were from the Ruaha ecosystem region, 72 (62.6%) were females, and the median age was 40 years old. Furthermore, eight study participants tested positive for RVFV by RT-qPCR, but near the limit of detection, and all had low calculated virus loads with cycle threshold (Ct) levels greater than 35. From these, six were detected in the dry season and two in the wet season. Still, of the eight RT-qPCR-positive participants, six had tested seronegative, one had tested positive for both IgM and IgG, and another one had tested positive for IgG only. While all eight patients had presented with fever and headache, only three were malaria positive. Among the acute/sub-acute cases of RVF (RT-qPCR and/or IgM positive) (n = 59), clinical signs were non-specific, but consistent with previous reports of RVF disease among hospitalized patients [6,66,67] (Table 2).

In the univariable analysis, recent exposure to RVFV (IgM positive only) was significantly associated with district (OR 3.0 Iringa rural; 95% CI 1.08–8.33), occupation (OR 0.19 Other; 95% CI 0.07–0.52), livestock keeping (OR 1.97; 95% CI 1.03–3.76), involvement in the slaughtering of livestock (OR 2.43; 95% CI 1.00–5.89), and milking animals (OR 2.12; 95% CI 1.03–4.34). However, no variables remained significant in the multivariable model.

Regarding overall seropositivity (IgG and/or IgM positive), in the univariable analyses, significant risk factors were age, occupation, livestock ownership, and slaughtering of livestock (Table 3). There was no association with gender, region, consumption of raw livestock products (blood and meat), consumption of meat, drinking milk, consumption of raw milk, season, handling aborted fetus material, and milking (Table 3). In the multivariable analysis, region (aOR 2.63 Iringa; 95% CI 1.26–5.50) and ownership of livestock (aOR 2.51; 95% CI 1.0–6.32) were significantly associated with overall seropositivity, and including the variable home slaughtering, improved model fit (Table 4).

### 3.2. Livestock Exposure to RVFV, Animal-Level Risk Factors, and Geographic Clustering

From the total of 4449 animals sampled, 103 from the Morogoro region were reported by owners to have previously been vaccinated against RVF, and this group had a seroprevalence of 33.0%, with one animal also being anti-RVFV IgM positive. These vaccinated animals were excluded from the subsequent analysis, and a total of 4346 animals were further analyzed, of which 67.1% were female, and 69.9% were ≥2 years old. Among the 193 herds, 132 had evidence of RVFV exposure, with a higher proportion of seropositive herds detected in the Kilombero ecosystem (Morogoro region 74/93 herds; 79.6%) compared to the Ruaha ecosystem (Iringa region 58/100 herds; 58.0%). Herd recent exposure (likely within the past 60–90 days), as indicated by anti-RVFV IgM detection in at least one animal, was higher in the Morogoro region (15/93 herds; 16.1%) than in the Iringa region (12/100 herds; 12.0%).

RVFV overall seroprevalence (IgG and/or IgM positive) in all livestock at an individual level was 9.57% [95% CI 8.71–10.48], with markedly higher seropositivity detected in the Morogoro region (16.8%) compared to the Iringa region (5.8%). Among the various livestock species, RVFV overall seroprevalence (IgG and/or IgM positive) was 15.3%, 3.8% and 5.7% in cattle, goats, and sheep, respectively. Recent exposure seroprevalence (IgM positive) was 1.1% [95% CI 0.76–1.50] and similar between regions. No RT-qPCR test resulted positive for livestock (Table 1). Spatial cluster analysis identified one statistically significant cluster of cases located within the Kilombero ecosystem (Figure 1), which included nine herds, in a 10.67 km radius with a relative risk (RR) of 5.77.

Univariable analyses for overall seroprevalence (IgG and/or IgM positive) in livestock indicated RVFV was associated with region, district, age group, season, and species (Table 5). In the multivariable model, it was associated with region, species, season, and age (Table 6). Livestock aged >2 years old had 4.97 (95% CI: 2.98–8.26) odds of exposure compared to animals aged <1 year old. The odds of exposure in cattle were 4.24 (95% CI: 2.99–6.02) while adjusting for age and region. Livestock in the Kilombero ecosystem had higher odds of being seropositive than those in the Ruaha ecosystem (aOR 2.81, 95% CI: 1.82–4.35).

### 3.3. Sentinel Animal Seroconversion Monitoring

**Figure 2:**
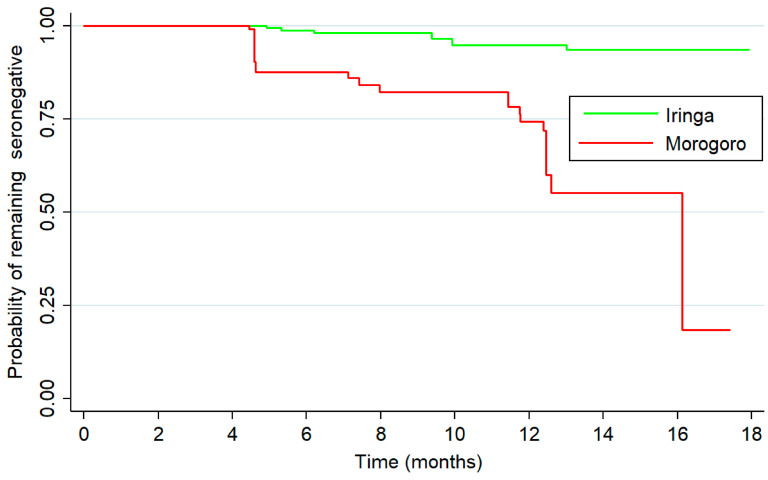
Kaplan–Meier survival estimates of sentinel animals in the Morogoro and Iringa regions. The lines indicate the probability of remaining seronegative in months for 328 animals monitored for seroconversion in the sentinel herd surveillance program between April 2016 and October 2017. The log-rank *p* < 0.0001.

A total of 43 herds, including 21 in the Ruaha ecosystem and 22 located in the Kilombero ecosystem, were enrolled for longitudinal monitoring as part of a sentinel animal (n = 328) surveillance program (Figure 1). At the end of the 18-month follow-up period, 16 herds had at least one animal that seroconverted (10 herds from Morogoro and 6 herds from the Iringa region) (Figure 2). The proportion of animals followed up until the end of the 18-month period was similar in the two regions (23.7% in Morogoro and 31.6% in Iringa).

### 3.4. RVFV Genome Detection and Blood Meal Analysis of Mosquitoes

A total of 23,949 mosquitoes were processed for further testing and were organized into 3463 pools of up to 25 mosquitoes by collection date, taxa, sex, and blood meal status for laboratory analyses (Table S1). Most catches (93%) were made during the rainy season. The majority of catches were from *Culex* spp. (including *Lutzia*, 47.5%) and *Anopheles gambiae* sl. (23.6%). Overall, 6.2% of mosquitoes were male, 87.7% were unfed females, and 6.1% were blood-fed females.

The RVFV genome was detected in a total of 22 pools, broken down as 7 pools of *Culex* spp., two pools of *Anopheles gambiae* s.l., 12 pools of *Anopheles pharoensis*, and 1 pool of *Mansonia uniformis*. These specimens were collected near flooded areas adjacent to the Mtera dam, an artificial water reservoir located in the Iringa region, in February and April 2018 (Figure 1).

For blood-engorged mosquitoes, 303 of 451 blood meals (67.2%) were identified across the study duration. Fourteen blood meals were from *Aedes* spp., nine from *Lutzia*, and the remainder from *Culex* spp. Cattle, dogs, and goats were heavily utilized hosts by a diversity of *Culex* mosquitoes (Table 7).

## 4. Discussion

This study provides a multi-year examination of interepidemic transmission of RVFV across human, animal, and vector populations in a region that is historically known to experience RVF outbreaks [43]. We demonstrate that, over a two-year period with no formal disease outbreaks declared in central Tanzania, RVFV was actively being transmitted between vectors, livestock, and people. The widespread ecological transmission of RVFV in the study region is evidenced by the large numbers of IgM-positive detections among both humans and livestock, RT-qPCR-positive human cases and mosquito pools, and the longitudinal detection of seroconversion in sentinel cattle herds. The lack of recognition of notable RVFV transmission activity involving livestock and humans underscores the difficulties in differentiating RVF from other acute-onset arboviral illnesses and highlights a critical gap in integrated One Health approaches to disease biosurveillance across this African region. The similarity of clinical signs of RVF and other arboviral diseases, especially among human patients, likely complicated the clinical identification of RVF, as the majority of laboratory-confirmed cases in this study had fever, malaise, headache, chills, joint pain, abdominal pain, and muscle pain, which are common to many tropical diseases.

Increasingly, longitudinal prospective studies of RVFV ecology and epidemiology have revealed that the classical paradigm of quiescence, followed by large region-wide outbreaks, may be only one component of the RVFV life cycle. Variability in interepidemic transmission observed over space and time (e.g., Tanzania [43,66,68,69,70,71], Uganda [32,60,72], Kenya [27,41,42,73], Sudan [9,10,74], Madagascar [73], and South Africa [49,50,51,75,76,77]), accentuates the need for tailored approaches that account for nuances of viral ecology coupled with active biosurveillance activities.

As a high-consequence zoonotic pathogen, RVFV exploits the connections between humans, animals, arthropod vectors, and the environment and poses a significant threat to global health and food security through the introduction of RVFV into previously naïve areas [35,78,79,80,81]. Simply put, collectively available data suggests that the epidemiology of Rift Valley fever is changing or at least more frequently presenting as endemic transmission in locally defined areas, without the explosive case-loads that have historically signaled an RVF “outbreak”. Thus, biosurveillance study designs centered on One Health approaches are essential to ensure that timely and appropriate public health and veterinary intervention measures can be taken.

Using this approach to understand the ecological drivers that maintain RVFV circulation during inter-epidemic periods, as well as those that tip the scales into large-scale outbreak situations, is critical for prioritizing meaningful preventative actions. Given our data demonstrating frequent livestock transmission and the ecological importance of cryptic cycles among livestock, and perhaps other wildlife as amplification hosts for ongoing virus replication and transmission among vectors and humans, this could be a key driver of endemic RVFV transmission at the population level. This study lays the groundwork for a more integrated biosurveillance program in Tanzania to identify geographic areas of localized transmission and populations associated with higher transmission risk and to assess the seasonality of active transmission among vectors, livestock, and humans.

Over the course of the study, recent infections were determined in humans, livestock, and mosquito vectors through the detection of IgM antibodies or RVFV genome in clinical specimens. The alignment of these results across human, livestock, and vector sampling advances the knowledge of RVFV ecology in our study area by helping bracket the timing of when exposures occurred. The detection of eight (8) RVFV low-level PCR-positive individuals and a larger number of persons (n = 52) positive for RVFV-specific IgM antibodies were strong indicators of recent and ongoing infection in humans [33,82]. These findings also interestingly coincided temporally with an overall increase in RVFV activity across East Africa more broadly at the given time, as evidenced by reports of livestock and human cases in Kenya and Uganda since January 2018 [83,84].

The detection of IgM antibodies in two children (a 5-year-old and a 7-year-old) from livestock or agricultural subsistence farming households further indicates the active circulation of RVFV during the study period across a spectrum of age groups and outside of any known large-scale outbreak. While the exact duration of RVFV IgM antibodies is not well characterized in humans, in animals, the median duration for anti-RVFV IgM antibodies is approximately two months [33,82,85] and approximately 2 to 4 months in limited human studies [86]. This suggests that exposures for these individuals occurred within the study period and likely just prior to presentation at rural health clinics. These two recent cases highlight a challenge in RVFV diagnostics in endemic settings, as both were RT-qPCR negative by the time of specimen collection. In this study, this finding was common with the detection of the RVFV genome occurring infrequently despite utilizing a sensitive, well-established assay [62] and suggests that in routine screening of ill patients in some areas, the detection of viremic patients may be challenging. In light of this, rapid IgM serology may serve as an important and underutilized diagnostic modality to track RVFV activity.

Interestingly, this study’s cohort of IgM-positive persons was detected in every month of the study period except for March and December, lending further support that transmission occurs during most of the year in this region. The finding that the majority of IgM+ and PCR+ human patients occurred during the dry season periods suggests that while rainfall likely plays a role in driving transmission, RVF does not necessarily occur only in association with the rainy season and the expected increases in vector activity and may be driven by direct contact transmission from viremic symptomatic animals infected via persistent low-level mosquito breeding at a localized scale where conditions may allow for ongoing RVFV transmission. In contrast, and serving to illustrate the complexity of RVFV ecology and the importance of local factors driving transmission, in January and February at the early stages of the 2018 rainy season, RT-qPCR-positive mosquito pools and one human case were detected at sampling sites and rural health clinics near a large water reservoir (Mtera Dam) in the Ruaha ecosystem area.

Among this cohort of study participants, typical significant risk factors associated with RVFV exposure in the univariable analyses included occupation factors such as livestock ownership (OR 2.4, 95% CI 152- 3.81), slaughtering of livestock (OR 1.97, 95% CI 1.00–3.89), and participating in pastoral animal grazing practices (OR 2.25, 95%CI 1.16–4.37). Interestingly, the location of the household was highly associated with seropositivity in this study, with persons living in the Iringa Rural District (Ruaha ecosystem) being 2.33 times more likely (OR 2.33, 95% CI 1.25–4.32) to be exposed to RVFV. Other factors that trended towards a positive association, but were not significant, with RVFV exposure included handling of aborted fetuses, milking of livestock, and being a male person. Multivariable analyses across all potential risk factors identified two strongly associated risk factors, including livestock ownership (aOR 2.63, 95%CI 1.26–5.50) and location of the household within the Ruaha ecosystem (Iringa District; aOR 2.51, 95%CI 1.00–6.32). Taken together, the risk factor analyses completed in this study did not identify any atypical factors driving what appears to be endemic and continuous circulation of RVFV in the study area.

As expected, contact with livestock (herding, milking, and slaughtering) tended to result in a higher risk of exposure as observed among pastoralists, as compared to other occupational groups and their respective risk of RVFV seropositivity. The higher seroprevalence in humans in the Ruaha ecosystem (especially the Iringa Rural District ) may be explained by the presence of suitable mosquito vectors and larger livestock populations. Higher numbers of susceptible animals would support robust RVFV amplification and increased vector maintenance/transmission of the virus, and suggest the presence of region-specific factors, which might influence inter-epidemic exposure of humans. RVFV transmission is known to vary due to fine-scale differences in various localized conditions, including climatic and environmental factors [87].

Detection of anti-RVFV IgM antibodies in livestock in both regions suggested an active and potentially sub-clinical infection in livestock populations, which might be connected to human cases, considering the intense human–livestock interaction during milking, slaughtering, and taking care of sick animals. Livestock positive for IgM were found at all three locations where PCR-positive mosquitoes were detected, and at two sites (Kimande and Idodi), RVFV-positive livestock, people, and mosquitoes were found (Figure 1). The overall seroprevalence in livestock reported here is lower compared to other studies on inter-epidemic seropositivity in Tanzania [88] and other parts of Africa [77,87,89] and was likely due to the inclusion of large numbers of sheep and goats, which have shorter life spans than cattle, in the livestock cohort. While adjusting for age and region, cattle had greater odds of exposure to RVFV than sheep and goats and suggests why the odds of seropositivity in animals aged more than 2 years were high compared to younger ones, as cattle are much longer lived in the study area.

The RT-qPCR detection of RVFV in humans and *Culex* spp. and *Anopheles* spp. mosquito pools in adjacent areas in early 2018 during the rainy season further supports the endemic transmission of RVFV in this region (Figure 1). Nonetheless, *Aedes* spp. mosquitoes were not commonly collected during this study. Since mosquito collections were performed alongside cross-sectional livestock surveillance and were not necessarily timed when *Aedes* are abundant, it is likely that the involvement of *Aedes* spp. mosquitoes is underrepresented in our dataset and was missed at the very beginning of the rainy season, as these mosquitoes emerged due to logistical constraints that prevented continuous mosquito surveillance activities during the entire study period. However, the RVFV genome detection in *Culex* and *Anopheles* species does suggest a role for these vectors in maintaining RVFV transmission among livestock and potentially humans during interepidemic periods. Unfortunately, detailed speciation of *Culex* spp. was not completed due to logistical constraints and thus limited insights into the exact species composition and contribution of specific vectors on the observed RVFV transmission ecology. Blood meal analyses (n = 449) revealed that the majority of captured engorged female mosquitoes had fed on cattle (n = 222), with the next most frequent detection being “unknown” (n = 146), suggesting a wide range of potential wildlife or other common domestic hosts such as chickens across the study regions. While not surprising, this finding highlights the potential of cross-species transmission of RVFV and other arboviruses from domesticated livestock to other species, as found in other countries [12,90,91]. Future studies should focus mosquito surveillance efforts more intentionally across seasons to capture any seasonal differences in mosquito community composition and the involvement of different vectors early and late into the rainy season [14].

## 5. Conclusions

In this study, RVFV transmission was evaluated among humans, livestock, and mosquito vectors in two regions of Tanzania, over a two-year period. The detection of RT-qPCR-positive mosquitoes and humans during the early rainy season (January) of 2018, multiple livestock and human IgM-positive cases, and frequent seroconversions in cattle in the absence of a reported outbreak suggests seasonal endemicity of RVFV in the study area. The paucity of *Aedes* spp. mosquito vectors and positivity of secondary mosquito vectors in the *Culex* and *Anopheles* genera also suggested that transmission cycles either started even earlier in the sampling seasons or were occurring continuously over this period. The detection of RVFV genomes in mosquitoes and humans during the dry season was particularly remarkable in this regard, lending combined evidence towards this region experiencing endemic transmission [32,40,41,42,43,44,45,46,47,48]. Livestock keeping, and specifically involvement in milking and slaughtering livestock, were risk factors highly associated with RVFV exposure in this region, as has previously been reported [17,92,93]. Among RVFV RT-qPCR-positive human patients, virus loads did not permit virus genome sequencing, so the exact molecular identity of the RVFV circulating during this time frame could not be established. However, taken together, these study results indicate that RVF is endemic in this area, and exposure risk remains an underreported health challenge to people and animals in these locations. The findings from this integrated One Health surveillance approach demonstrate the value added from collecting concurrent human, livestock, and vector data through integrated One Health surveillance. Early season detections of virus circulation were achieved, and preliminary transmission-relevant connections were made between mosquito vector species, cattle, and high-risk activities associated with livestock keeping. Active surveillance and evidence-based control strategies, including community health education and risk mitigation education, enhanced and improved livestock keeping best practices to avoid virus contact, and integrated human and animal vaccination campaigns, when available, will likely reduce the impact of this significant health threat. Tackling the challenges of RVFV across Africa will require a concerted effort on the part of the human and veterinary sectors to lead to meaningful and impactful changes to save the lives and improve the livelihoods of the people and their animals in endemic areas.

## Figures and Tables

**Table 1:** Overview of laboratory test results for humans, livestock (individual and herd level), and mosquito vector samples by region ^†^.

Population	Sampled with Complete Data	Laboratory Test	Prevalence% (Positive/Total Tested)		
			Ruaha Ecosystem Iringa Region	Kilombero Ecosystem Morogoro Region	Total
Humans (n)	1385	ELISA IgG and/or IgM *	10.46% (78/746)	5.79% (37/639)	8.30% (115/1385)
		ELISA IgG	8.71% (65/746)	5.16% (33/639)	7.08% (98/1385)
		ELISA IgM	4.96% (37/746)	2.35% (15/639)	3.75% (52/1385)
		RT-qPCR	0.40% (3/746)	0.78% (5/639)	0.58% (8/1385)
Cattle (n)	2300	ELISA IgG and/or IgM *	9.20% (116/1261)	22.62% (235/1039)	15.26% (351/2300)
		ELISA IgG	8.58% (106/1236)	22.62% (230/1017)	14.91% (336/2253)
		ELISA IgM	1.28% (11/858)	1.19% (10/838)	1.24% (21/1696)
		RT-qPCR	0.00% (0/357)	0.00% (0/270)	0.00% (0/627)
Goats (n)	1193	ELISA IgG and/or IgM *	2.64% (23/871)	6.83% (22/322)	3.77% (45/1193)
		ELISA IgG	2.20% (19/863)	6.62% (21/317)	3.39% (40/1180)
		ELISA IgM	0.70% (4/571)	0.38% (1/261)	0.60% (5/832)
		RT-qPCR	0.00% (0/259)	0.00% (0/117)	0.00% (0/376)
Sheep (n)	956	ELISA IgG and/or IgM *	2.83% (16/565)	9.72% (38/391)	5.65% (54/956)
		ELISA IgG	2.14% (12/562)	8.31% (32/385)	4.65% (44/947)
		ELISA IgM	0.89% (4/449)	1.80% (6/333)	1.28% (10/782)
		RT-qPCR	0.00% (0/160)	0.00% (0/130)	0.00% (0/290)
Total livestock (cattle, goats, and sheep) (n)	4449	ELISA IgG and/or IgM *	5.75% (155/2697)	16.84% (295/1752)	10.11% (450/4449)
		ELISA IgG	5.15% (137/2661)	16.46% (283/1719)	9.59% (420/4380)
		ELISA IgM	1.01% (19/1878)	1.19% (17/1432)	1.09% (36/3310)
		RT-qPCR	0.00% (0/776)	0.00% (0/517)	0.00% (0/1293)
Livestock (herds)	193	ELISA IgG and/or IgM *	58.00% (58/100)	79.57% (74/93)	68.39% (132/193)
		ELISA IgG	56.00% (56/100)	79.57% (74/93)	67.36% (130/193)
		ELISA IgM	12.00% (12/100)	16.13% (15/93)	13.99% (27/193)
		RT-qPCR	0.00% (0/50)	0.00% (0/46)	0.00% (0/96)
Mosquito vectors (pools)	3463	RT-qPCR	1.73% (22/1272)	0.00% (0/2191)	0.64% (22/3463)

^†^ Note: Not all specimens were available for both IgG and IgM testing. * Overall seroprevalence.

**Table 2:** Clinical characteristics of human acute/sub-acute RVF cases (RT-qPCR and/or IgM positive).

Clinical Sign	RT-qPCR Positive (n) ^1^	ELISA IgM Positive ^1^	RT-qPCR and ELISA IgM Positive ^1^	Total Acute/ Sub-Acute Cases (n) ^1^	Affected Proportion (%)
Fever	7	51	1	59	59/59 (100.0)
Chills	6	40	1	47	47/59 (79.7)
Malaise	6	40	0	46	46/59 (78.0)
Headache	7	38	1	46	46/59 (78.0)
Joint pain	6	34	0	40	40/59 (67.8)
Abdominal pain	5	32	1	38	38/59 (64.4)
Muscle pain	5	29	1	35	35/59 (59.3)
Inappetence	2	26	0	28	28/59 (47.5)
Cough	2	18	0	20	20/59 (33.9)
Dizziness	4	12	0	16	16/59 (27.1)
Eye pain	2	12	0	14	14/59 (23.7)
Vomiting	2	8	0	10	10/59 (16.9)
Throat pain	1	8	0	9	9/59 (15.3)
Diarrhea	2	7	0	9	9/59 (15.3)
Stiff neck	1	5	0	6	6/59 (10.2)
Rash	0	2	0	2	2/59 (3.4)
Bleeding	0	1	0	1	1/59 (1.7)
Convulsion	0	0	0	0	0/59 (0.0)

^1^ Number of cases with a clinical characteristic and diagnostic test result.

**Table 3:** Univariable analyses of risk factors associated with RVFV overall seropositivity (IgG and/or IgM positive) in humans.

Variable	Category	Seropositive% (n)	OR	95% CI	***p*** **-Value**
Region	Kilombero ecosystem (Morogoro region)	5.79 (639)	r		
	Ruaha ecosystem (Iringa Region)	10.46 (746)	1.69	0.82–3.46	0.152
District	Kilombero	6.07 (461)	r		
	**Iringa rural**	**13.71 (496)**	**2.33**	**1.25–4.32**	**0.008**
	Kilolo	4.0 (250)	0.64	0.26–1.56	0.323
	Ulanga	5.06 (178)	0.83	0.30–2.25	0.707
Age	≤10 years	2.65 (151)	r		
	**>10 years**	**9.00 (1234)**	**3.28**	**1.19–9.04**	**0.022**
Occupation	Peasant	9.84 (894)	r		
	**Pastoralist**	**25 (64)**	**2.25**	**1.16–4.37**	**0.016**
	Other *	**2.64 (417)**	**0.23**	**0.12–0.43**	**<0.001**
Livestock ownership	No	6.96 (1077)	r		
	**Yes**	**13.16 (304)**	**2.4**	**1.52–3.81**	**<0.001**
Slaughters livestock for home consumption or sales	No	6.46 (356)	r		
	**Yes**	**10.20 (245)**	**1.97**	**1.00–3.89**	**0.049**
Consumes raw livestock product	Yes	7.70 (13)	r		
	No	10.37 (868)	1.46	0.18–11.68	0.723
Eats livestock meat	No	5.38 (186)	r		
	Yes	11.65 (695)	1.54	0.72–3.27	0.262
Milks livestock	No	9.70 (629)	r		
	Yes	11.90 (252)	1.66	0.97–2.85	0.067
Drinks livestock milk	No	7.44 (390)	r		
	Yes	12.63 (491)	1.36	0.80–2.32	0.258
Boils livestock milk before drinking	Yes	10.55 (455)	r		
	No	10.09 (426)	1.34	0.81–2.23	0.254
Season	Wet	8.44 (462)	r		
	Dry	8.23 (923)	1.2	0.79–1.83	0.394
Gender	Female	7.50 (960)	r		
	Male	10.11 (425)	1.42	0.95–2.12	0.088
Handling aborted fetuses	No	9.89 (637)	r		
	Yes	11.48 (244)	1.81	0.99–3.31	0.056

n = count; OR = Odds ratio; CI = Confidence interval; r = Reference level; Other * = Formal employment, children, and students; Ruaha ecosystem (Iringa region; Iringa and Kilolo districts); Kilombero ecosystem (Morogoro region; Kilombero and Ulanga districts).

**Table 4:** Multivariable analyses of risk factors associated with RVFV overall seropositivity (IgG and/or IgM positive) in humans.

Variable	Category	aOR	95% CI	*p*-Value
Region	Kilombero ecosystem (Morogoro region)	r		
	**Ruaha ecosystem** **(Iringa region)**	**2.63**	**1.26–5.50**	**0.01**
Livestock ownership	No	r		
	**Yes**	**2.51**	**1.00–6.32**	**0.05**
Home slaughtering	No	r		
	Yes	0.74	0.27–2.05	0.564

aOR = Adjusted odds ratio; CI = Confidence interval; r = Reference level.

**Table 5:** Univariable analyses of livestock risk factors associated with RVFV overall seropositivity (IgG and/or IgM positive) in livestock.

Variable	Category	Seropositive% (n)	OR	95% CI	*p*-Value
Region	Ruaha ecosystem (Iringa region)	5.74 (2697)	r		
	**Kilombero ecosystem** **(Morogoro region)**	**15.82 (1649)**	**3.51**	**2.36–5.23**	**<0.001**
Species	Sheep	5.50 (946)	r		
	**Cattle**	**14.47 (2212)**	**3.65**	**2.61–5.12**	**<0.001**
	Goats	3.70 (1188)	0.81	0.52–1.25	0.334
Sex	Male	9.3 (1426)	r		
	Female	9.7 (2916)	1.13	0.89–1.44	0.32
Age	Less than 1 year	4.9 (360)	r		
	1–2 years	4.72 (868)	1.31	0.72–2.38	0.37
	**More than 2 years**	**11.62 (3039)**	**3.72**	**2.27–6.12**	**<0.001**
Season	Wet	5.5 (2125)	r		
	**Dry**	**13.46 (2221)**	**2.88**	**1.89–4.39**	**<0.001**
Origin	Without	13.52 (170)	r		
	Within	9.43 (4167)	0.73	0.42–1.26	0.252

n = count; OR = odds ratio; CI = Confidence interval; r = Reference level.

**Table 6:** Multivariable analyses of risk factors associated with RVFV overall seropositivity (IgG and/or IgM positive) in livestock.

Variable	Category	aOR	95% CI	*p*-Value
Age	Less than 1 year	r		
	1–2 years	1.46	0.80–2.69	0.218
	**>2 years**	**4.97**	**2.98–8.26**	**<0.001**
Species	Sheep	r		
	**Cattle**	**4.24**	**2.99–6.02**	**<0.001**
	Goats	0.88	0.57–1.38	0.585
Region	Ruaha ecosystem (Iringa region)	r		
	**Kilombero ecosystem** **(Morogoro region)**	**2.81**	**1.82–4.35**	**<0.001**
Season	Wet	r		
	**Dry**	**1.78**	**1.14–2.77**	**0.011**

aOR = adjusted odds ratio; CI = Confidence interval; r = Reference level.

**Table 7:** Blood meal sources of engorged mosquitoes captured in Iringa, Kilombero, and Ulanga districts, 2016–2018.

Population	Blood Meal Source					
	Cattle	Cattle + Goat	Dog	Goat	Not Identified	Total
*Aedes aegypti*	0	0	0	0	2	2
*Aedes* spp.	3	0	1	0	8	12
*Culex pipiens/quinquefasciatus*	96	0	32	10	58	196
*Culex poicilipes*	21	0	14	0	7	42
*Culex* spp.	96	1	12	9	72	190
*Lutzia tigripes*	6	0	2	0	1	9
**TOTAL**	222	1	61	19	148	451

## Data Availability

The raw data supporting the conclusions of this article will be made available by the authors on request.

## Supplementary Materials

The following supporting information can be downloaded at: https://www.mdpi.com/article/10.3390/v17101329/s1, File S1: Structured questionnaire for RVFV risk factor assessment in humans. Table S1: Summary of mosquito species collected across the study area by region and year of sampling.

## Abbreviations

The following abbreviations are used in this manuscript:

AIC	Akaike information criteria
aOR	Adjusted odds ratio
CI	Confidence interval
ELISA	Enzyme-linked immunosorbent assay
OD	Optical density
OR	Odds ratio
RT-qPCR	Reverse Transcription Quantitative Polymerase Chain Reaction
RVF	Rift Valley fever
RVFV	Rift Valley fever virus
