# Supplementary material for: Rift Valley Fever Virus Transmission During an Unreported Outbreak Among People and Livestock in South-Central Tanzania
Source: Viruses. 2025 Sep 30;17(10):1329. doi: 10.3390/v17101329 (PMC12567855; doi:10.3390/v17101329)
Supplement: Supplementary file 1 [file viruses-17-01329-s001.zip › File S1.pdf]

Household Survey for Zoonotic Diseases

Interviewer's name: ______________________Today's date (DDMMYYYY): _ __/__ __/201__

Participant ID #: _ _ __ __ __ __ __ __

Section A: General participant Information

A1. How long have you lived in your village? [____] Years and/or [____] Months

A2. How many adults and kids live with you? [____] Adults [____] Children (0-18years)

A3. What are the occupations of other members of your family? Select all that apply.

Raising Animals [ ]
Agriculture (subsistence) [ ]
Agriculture (cash crops or market gardens) [ ]
Agro-pastoralist [ ]
Pastoralist [ ]
Merchant or Trade [ ]
Work in Park or Forest Reserve [ ]
Mining [ ]
Hunting [ ]
Other [ ], Specify____

Section B: Nutrition/Water

B1. Do you slaughter livestock/poultry/chicken for home consumption or selling meat at your homestead?

Yes [ ] Don't Know [ ]
No [ ] No Answer [ ]

B2. How frequently do you slaughter and/or butcher livestock? *(Specify animal type and frequency)*

Animal	Everyday	How many times /week	How many times / Month	How many times / Year	Never	Once in the past 12 mo
Goat						
Cow						
Other (specify)						

B3. Who is involved in the slaughtering? *[Mark all that apply]*

Head of the household [ ] Don't know [ ]
Young Men in the household [ ] No Answer [ ]
Any Adult Person Present [ ] Other [ ]*Specify:*______________

B4. Are there any animal parts or blood that are consumed raw immediately after slaughtering of an animal at your household?

Household Survey for Zoonotic Diseases

Interviewer's name: ______________________Today's date (DDMMYYYY): _ __/__ __/201__

Participant ID #: _ _ __ __ __ __ __ __

Yes	[ ] *If yes, what:*______________	Don't Know	[ ]
No	[ ]	No Answer	[ ]

B5. How frequently do you consume raw animal parts or blood from these animals? *(Specify animal type and frequency)*

Animal	Everyday	How many times / week	How many times / Month	How many times /Year	Never	Once in the past 12 mo
Goat						
Sheep						
Cow						

B6. Who normally inspects the meat when you do a home slaughter?

- Livestock Extension Officer [ ]
- Health Officer [ ]
- Agricultural Extension Officer [ ]
- No One [ ]
- Others [ ] *Specify*: ____________________________

B7. How frequently do you eat domestic animal meat?

Type of meat	Everyday	How many times / week	How many times /Month	How many times / Year	Once in the past 12 mo	Never

B8. If an animal dies would your family members usually take the meat from the animal for home consumption or sell?

Home Consumption	[ ]	Don't Know	[ ]
Sell the Meat	[ ]	Other	[ ]*Specify:*______________

B9. We understand that eating wild animals may be uncommon. However, do you or anyone you know in the community sometimes eat meat from wild animals? What kind of wild animals **do you or others** eat? *(Specify types of animals –species if possible and frequency)*

Self: Y[ ], N[ ] Others: Y[ ], N[ ]

Animal Type	Everyday	Weekly	Monthly	Yearly	Never
	[ ]	[ ]	[ ]	[ ]	[ ]
	[ ]	[ ]	[ ]	[ ]	[ ]

Household Survey for Zoonotic Diseases

Interviewer's name: ______________________Today's date (DDMMYYYY): _ __/__ __/201__

Participant ID #: _ _ __ __ __ __ __ __

[ ] [ ] [ ] [ ] [ ]
[ ] [ ] [ ] [ ] [ ]

B10. Where can people get wild animal meat/products? *(Check all that apply and specify type of animals)*

Hunting [ ] Friend [ ] Don't know [ ]
Vermin Control [ ] Other [ ]
Market [ ] *Specify:* ________________________

B11. How frequently do you milk livestock?

	Everyday	How many times /week	How many times / Month	How many times /Year	Never	Once in the past 12 mo
Cow						
Goat						
Other						

B12. Who is responsible for milking cattle and/or goats in your household? *[Mark all that apply]*

Women [ ] Don't know [ ]
Young Boys [ ] No Answer [ ]
Young Girls [ ] Other [ ]
Head of Household [ ] *Specify:*_____________________________________

B13. How frequently do you drink milk from your animals?

	Everyday	How many times / week	How many times / Month	How many times / Year	Never	Once in the past 12 mo
Cow						
Goat						
Sheep						

B14. How frequently do you boil milk before drinking it?

Everyday [ ] Never [ ]
How many Times / Week [ ] Only Once in the Past [ ]
How many Times / Month [ ] 12 Months [ ]
How many Times /Year [ ] Other [ ]
Specify: ____________________________________

B15. What do you do to control mosquitoes in your home? Please check all that apply.

Spray [ ] Bed Nets [ ]

Household Survey for Zoonotic Diseases

Interviewer's name: ______________________Today's date (DDMMYYYY): _ __/__ __/201__

Participant ID #: _ _ __ __ __ __ __ __

Overturn Standing Water [ ] Nothing [ ]
Smoke [ ] Other [ ]
*Specify:*___________________________________

Section C: Zoonotic Disease

C1. Do you think it's possible for diseases to be transmitted to people from animals?
Yes[ ] No[ ] Don't Know[ ]

C2. If yes, please specify which animals and what types of diseases

____
____
____
____

Section D: Livestock (*If the household does not have any animals then skip to section E).*

D1. How many of each of the following animals do you have?

Cattle	[ ]	Chickens	[ ]
Goats	[ ]	Cats	[ ]
Sheep	[ ]	Dogs	[ ]
Pigs	[ ]	Other	[ ]*Specify:*__________________________

D2. Have you recently (past 1-6 Months) introduced a new animal/animals to your herd/flock?
[___] Yes [___] No, If yes [Please *specify animal species :*__________________________

D3. If the answer to Question D2 is yes, what were the sources of the new animal(s)?

Animal Auction	[ ]
Gift	[ ]
Bridal Price	[ ]
Other	[ ] *Specify:*__________________________

D4. How are your livestock kept? (1=Intensive/housing, 2=Free-Ranging, 3=Grazing with supervision/guarding, 4=other)

Cattle	[ ]	Chickens	[ ]
Goats	[ ]	Cats	[ ]
Sheep	[ ]	Dogs	[ ]
Pigs	[ ]	Other	[ ]

*Specify:*____

D5. If the animals are grazed, what areas are used?
Inside Forest [ ] Open Grass [ ]

Household Survey for Zoonotic Diseases

Interviewer's name: ______________________Today's date (DDMMYYYY): _ __/__ __/201__

Participant ID #: _ _ __ __ __ __ __ __

Lands at the Forest Edge	[ ]	Other	[ ]
Farms	[ ]	*Specify:*______________________________	

D6. In the case of an animal giving birth, who is responsible for assisting? *[Select all that apply]*

Anybody around when event occurs	[ ]	Men Only	[ ]
Herdsman/boy	[ ]	Don't Know	[ ]
Head of Household	[ ]	No Answer	[ ]
Adults Only	[ ]		

D7. In the past 12 months have you experienced any recurrent abortions in your herd/flock? Which animals? (Recurrent = two or more in succession) *If the answer is NO, skip to Question F13.*

	Yes	No	Don't Know
Cattle	[ ]	[ ]	[ ]
Goats	[ ]	[ ]	[ ]
Sheep	[ ]	[ ]	[ ]
Pigs	[ ]	[ ]	[ ]
Cats	[ ]	[ ]	[ ]
Dogs	[ ]	[ ]	[ ]
Other	[ ]	[ ]	[ ] *Specify:* __________________________

D8. In the case of abortion in pregnant animals, what do you do with the aborted fetus? *[Select all that apply] , Please don't read the options given below*

Throw Away in Bush	[ ]
Dig a hole in the ground, place fetus and cover w/soil	[ ]
Feed to dogs/cats in raw form	[ ]
Feed to dogs/cats after boiling	[ ]
Don't Know	[ ]
No Answer	[ ]
Other	[ ] *Specify*: ___________________

D9. What was done to animals that experienced recurrent abortions? *Please don't read the options given below*

Sold to Animal Auction	[ ]	Left in the Herd/flock	[ ]
Sold to Butcher in Village	[ ]	Slaughtered at Home	[ ]
Sold to Villagers	[ ]	Other	[ ] *Specify*: __________

D10. Has there been an animal disease in the past that led many pregnant animals (goats, sheep and/or cattle) to abort in your herd? *If the answer is NO, skip to Question D12.*

Yes	[ ]*If yes, what:*__________	Don't Know	[ ]
No	[ ]	No Answer	[ ]

Household Survey for Zoonotic Diseases

Interviewer's name: ______________________Today's date (DDMMYYYY): _ __/__ __/201__

Participant ID #: _ _ __ __ __ __ __ __

D11. Did the disease also occur in other herds within the village or in the neighboring villages?

Yes	[ ]	*If yes, what:*__________	Don't Know	[ ]
No	[ ]		No Answer	[ ]

D12. Has there been an animal disease that led to many newborn and/or young animals to die in your herd?

Yes	[ ]	*If yes, what:*__________	Don't Know	[ ]
No	[ ]		No Answer	[ ]

D13. Did the disease also occur in other herds within the village or in the neighboring villages?

Yes	[ ]	*If yes, what:*__________	Don't Know	[ ]
No	[ ]		No Answer	[ ]

D14. If an animal is terminally or acutely ill, will you slaughter it for meat consumption at home or sell it before it dies?

Slaughter	[ ]	Don't Know	[ ]	
Sell	[ ]	Other	[ ]	*Specify:* __________________________

Interviewer's extra notes and comments:

____

____

____

____

____

____

____

____

____

Signature of Interviewer: ______________________________ Date: ______/_____/201___

Signature of Supervisor: _______________________________ Date: ______/_____/201___

1st Data entry by: _____________________________________ Date: _____/_____/201___

2nd Data entry by: _____________________________________Date: _____/_____/201___
