# Supplementary material for: Rift Valley Fever Virus Transmission During an Unreported Outbreak Among People and Livestock in South-Central Tanzania
Source: Viruses. 2025 Sep 30;17(10):1329. doi: 10.3390/v17101329 (PMC12567855; doi:10.3390/v17101329)
Supplement: Supplementary file 1 [file viruses-17-01329-s001.zip › Table S1.pdf]

Table S1. Summary of mosquito species collected across the study area by region and year of sampling.

Year/ Species	RUAHA ECOSYSTEM			KILOMBERO ECOSYSTEM			TOTAL			Grand Total
	Males	Unfed Females	Fed Females	Males	Unfed Females	Fed Females	Males	Unfed females	Fed females	
**2016**	**106**	**688**	**97**	**242**	**971**	**95**	**348**	**1659**	**192**	**2199**
Aedes species	2	8	0	0	0	0	2	8	0	10
Anopheles coustani	2	135	45	0	32	18	2	167	63	232
Anopheles funestus	1	65	3	1	74	1	2	139	4	145
Anopheles gambiae sl.	1	15	0	6	31	4	7	46	4	57
Anopheles pharoensis	1	59	24	0	1	0	1	60	24	85
Anopheles squamosus	0	7	1	1	251	45	1	258	46	305
Coquilettidia species	4	12	0	2	47	0	6	59	0	65
Culex species	95	377	21	132	352	19	227	729	40	996
Mansonia africana	0	2	0	3	87	3	3	89	3	95
Mansonia uniformis	0	8	3	97	96	5	97	104	8	209
**2017**	**191**	**2533**	**276**	**643**	**5334**	**507**	**834**	**7867**	**783**	**9484**
Aedes species	10	13	1	3	24	3	13	37	4	54
Anopheles coustani		231	47	18	935	173	18	1166	220	1404
Anopheles funestus	19	119	12	37	385	5	56	504	17	577
Anopheles gambiae sl.	52	869	114	59	1188	132	111	2057	246	2414
Anopheles pharoensis	4	189	26	0	44	7	4	233	33	270
Anopheles squamosus	0	0	2	1	131	10	1	131	12	144
Anopheles ziemanni	0	0	0	0	9	3	0	9	3	12
Coquilettidia species	0	8	0	3	127	8	3	135	8	146
Culex species	101	992	66	517	2174	141	618	3166	207	3991
Mansonia africana	5	68	6		114	8	5	182	14	201
Mansonia uniformis	0	44	2	5	203	17	5	247	19	271
**2018**	**148**	**4551**	**152**	**147**	**6924**	**344**	**295**	**11475**	**496**	**12266**
Aedes species	0	36	8	0	62	4	0	98	12	110
Anopheles coustani	0	1		2	832	66	2	833	66	901
Anopheles funestus	1	539	9	22	257	5	23	796	14	833
Anopheles gambiae sl.	145	2179	114	8	675	49	153	2854	163	3170
Anopheles pharoensis	0	0	0	0	4	1	0	4	1	5
Anopheles squamosus	0	1	1	0	2	0	0	3	1	4
Anopheles wellcomei	0	11	0	0	0	0	0	11	0	11
Anopheles ziemanni	0	10	4	0	0	0	0	10	4	14
Coquilettidia species	0	0	0	1	64	0	1	64	0	65
Culex species	2	1736	12	113	4330	198	115	6066	210	6391
Mansonia africana	0	20	4		219	8	0	239	12	251
Mansonia uniformis	0	18		1	479	13	1	497	13	511
**Grand Total**	**445**	**7772**	**525**	**1032**	**13229**	**946**	**1477**	**21001**	**1471**	**23949**
